# A reconfigurable hardware infrastructure for evaluating PUFs in non-volatile memories

**DOI:** 10.1016/j.ohx.2026.e00809

**Published:** 2026-07-13

**Authors:** Florian Frank, Stefan Katzenbeisser

**Affiliations:** Chair of Computer Engineering, University of Passau, Innstraße 43, 94032 Passau, Germany

**Keywords:** Physical Unclonable Function (PUF), Field Programmable Gate Array (FPGA), Printed Circuit Board (PCB)

## Abstract

Physical Unclonable Functions (PUFs) are widely recognized for deriving strong cryptographic keys from inherent manufacturing variations in hardware. A popular class of PUFs is extracted from memory modules already integrated into computing systems, offering a cost-efficient method for generating strong cryptographic keys. Various techniques exist for extracting PUF responses from such memory modules, including repeated row activation and exploitation of charge leakage effects, known as row hammering, variations in supply voltage, intentional violations of memory timing specifications, as well as the use of random startup values and data retention characteristics. To perform such experiments, especially on novel non-volatile memory modules, a dedicated measurement ecosystem is required, which is presented in this work. The proposed setup uses a custom PCB to connect different types of memories to reconfigurable hardware. An optimized hardware design was developed and deployed on an FPGA, enabling the execution of PUF experiments on various memory modules. The system allows for dynamic adjustment of timing specifications and voltage levels, even during experiment execution. Finally, a program is provided that schedules the experiments, retrieves the results, and enables persistent storage and evaluation methods.

## Graphical abstract

**Figure:**
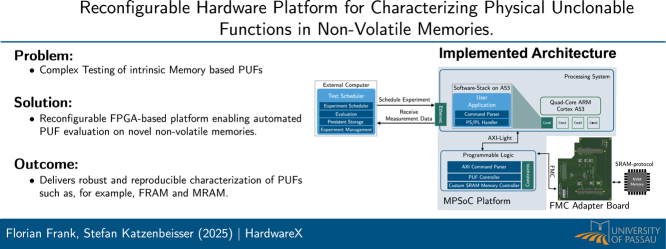

## Specifications table

**Table:** 

Hardware name	*Reconfigurable Memory-PUF Evaluation Platform*

Subject area	*Engineering and material science*

Hardware type	*Electrical engineering and computer science*

Closest commercial analog	*Antmicro Memory Test Platform*^a^*(price on request); SoftMC*^b^*(open-source, free); DRAM Bender*^c^*(open-source, free).*

Open source license	*Apache 2.0 (Software) and CERN OHL v2-P (Hardware)*

Cost of hardware	*The PCB adapter board (including components) costs $208, and the required MPSoC development board costs $3234 (prices as of December 2025).*

Source file repository	*https://doi.org/10.5281/zenodo.19205756*

a https://antmicro.com/platforms/memory-testing-platforms/.

b https://github.com/CMU-SAFARI/SoftMC.

c https://github.com/CMU-SAFARI/DRAM-Bender.

## 1 Hardware in context

Traditional memory technologies such as DRAM, SRAM, and NAND flash face growing limitations in access times, scalability, and energy efficiency. In response, emerging Non-Volatile Memories (NVMs), including Ferroelectric Random Access Memory (FRAM), Magnetoresistive Random Access Memory (MRAM), and Resistive Random Access Memory (ReRAM), have been developed to address these challenges [1]. Beyond their primary role as storage devices, memory modules can be used to extract hardware fingerprints, so-called Physical Unclonable Function (PUF) responses. These can, for example, be used as keys integrated into cryptographic applications built on top without requiring additional hardware for key extraction.

Legacy memory technologies already demonstrate such capabilities. For instance, DRAM suffers retention effects when the regular refresh is disabled, leading to bit-flips usable as hardware fingerprints [2]. Furthermore, such bit-flips can also be induced by performing row hammering experiments [3], in which physically adjacent DRAM rows are repeatedly activated to generate bit-flips through charge leakage effects similar to DRAM retention PUFs. Furthermore, SRAM-based implementations utilize device-unique startup values as responses [2], [4].

To extend these low-cost hardware security primitives to emerging non-volatile memories, similar experiments must be performed on these memory technologies, including intentional violations of the memory’s timing specifications, row hammering, or power supply manipulation. Executing these experiments demands a flexible measurement setup, specifically tailored for these use cases, yet flexible enough to allow the evaluation of different memory modules. Implementing such a setup is challenging, as it requires highly precise timing control as well as control over supply voltage levels of various memory modules.

Moreover, performing these experiments is time-consuming because typically the entire memory space is evaluated based on various configuration parameters including initialization values and timing parameters, resulting in a complex parameter space, where each configuration is referred to as a challenge. Therefore, the automatic scheduling of these experiments is essential to ensure an efficient and reproducible execution of experiments. To the best of our knowledge, there is currently no hardware platform that meets all of these requirements.

To address this gap, we introduce a Multi-Processor System-on-Chip (MPSoC)-based implementation featuring a custom memory controller implemented on the Field-Programmable Gate Array (FPGA) that is part of the MPSoC. Memory modules are connected via a custom-designed adapter through the board’s FPGA Mezzanine Card (FMC) interface, which ensures physical compatibility and performs logic-level conversion between the FPGA’s I/O pinout and the memory device. This design supports interfacing with memory modules that provide a parallel interface with up to 24-bit address width and a 16-bit data bus. It allows dynamic configuration of address and data bus widths, as well as fine-grained adjustment of timing parameters at runtime without requiring reconfiguration, with a timing resolution of 2.5 ns. Furthermore, the power supply can be fully decoupled and configured to support logic levels ranging from 1.2 V to 3.6 V. In addition, the project includes a second microcontroller-based implementation that supports Serial Peripheral Interface (SPI)-based memory modules. By offering parallel and SPI-based interfaces, the system can support the vast majority of commercially available NVMs. The hardware and software setup provides network interfaces that enable remote configuration of the experiments, as well as seamless readout and analysis of the collected data. Our platform also enables robustness tests under various environmental conditions, such as temperature variations and aging. To this end, it provides control over a climate chamber with a granularity of 0.1 °C, as demonstrated in our previous work [5]. Moreover, only the memory under test needs to be exposed to environmental changes, rather than the entire test setup, using extension cable compatible with our hardware.

In summary, our setup enables researchers to systematically evaluate complex parameter spaces of potential PUFs in emerging memory technologies, while providing a platform for reproducible results, simple interfacing of memory modules, straightforward configuration, and significant time savings through automated test execution.

## 2 Hardware description

The contributions presented in this work consist of three components that together form a setup capable of performing various PUF experiments on a selection of memory modules. It further enables the scheduling of experiments, their persistent storage, and subsequent analysis. These components are defined as follows:

• A generic hardware adapter for connecting a wide range of memory modules to an MPSoC platform. This adapter not only maps the pinout of different memory modules to a generic interface, but further allows adjustments of different logical voltage levels, the connection with external power supplies, while using a Low-Noise RF Printed Circuit Board (PCB) layout.
• A highly flexible and customizable memory evaluation platform implemented on an FPGA, adopting a runtime-adjustable Static Random-Access Memory (SRAM) protocol and the hardware-based implementation of various PUF experiments.
• A software framework for automated scheduling of memory experiments, with persistent result storage enriched with metadata. It furthermore enables the evaluation of collected results. Additionally, this scheduler offers endpoints for integration with a graphical user interface, allowing for seamless test scheduling and real-time monitoring.

In the following sections, a detailed description of these components is provided, beginning with the physical interface, followed by the implementation of the FPGA hardware design and the required software components.

### 2.1 Uniform memory interface

The test setup presented in this work enables a consistent and efficient evaluation of various memory modules that offer an SRAM-compatible interface. Since the memories under test differ in packaging, address, data widths, and electrical characteristics, a flexible adapter is required to support quick and seamless swapping of memory modules, while allowing the interfacing of these modules with either FPGA-based or microcontroller-based memory controllers, as well as with external devices such as power supplies.

To meet these requirements, adapter boards with a uniform interface are designed for each memory model, encapsulating the specific package and ensuring compatibility with the utilized memory controller. These adapters, fabricated as custom PCBs, adopt the specific packaging and wiring of each memory while providing a uniform interface on the opposite side. This interface is compatible with the STM32F429I-DISC0 board, which was used as the initial measurement platform. However, because of its limited flexibility and especially due to its reduced timing resolution, a new hardware and software design was developed targeting MPSoC architectures.

In Fig. 1(a), the MPSoC adapter to connect to the ZCU102 evaluation board via an FMC adapter is shown. Stacked on top is an adapter adopting the STM32 interface, which offers an interface for TSOP-48 packaged memory modules. Fig. 1(b) shows the PCB design of the MPSoC adapter board. At the bottom of the board, the FMC connector is shown, which interfaces with the FMC port (J5) of the ZCU102. The two vertical 2 × 32 pin headers implement the STM32 interface. In the lower-right corner, a PCIe ×1 extension slot is provided, allowing the integration of additional extension cards, for example, to implement VADJ adjustment and detection according to the ANSI/VITA 57.1 FMC specification [6], which is not included in the current board revision. Dedicated pin headers are used for debugging purposes, as well as for ground and power connections to external devices. Our adapter board uses the FMC low-pin-count subset, enabling compatibility with more cost-efficient MPSoCs, such as the Digilent ZedBoard.Fig. 1(a) Stacked PCBs providing a uniform interface to the ZCU102 evaluation board. (b) FMC adapter board matching the wiring and electrical characteristics between the memory module and the ZCU102.Fig. 1

**(a):**
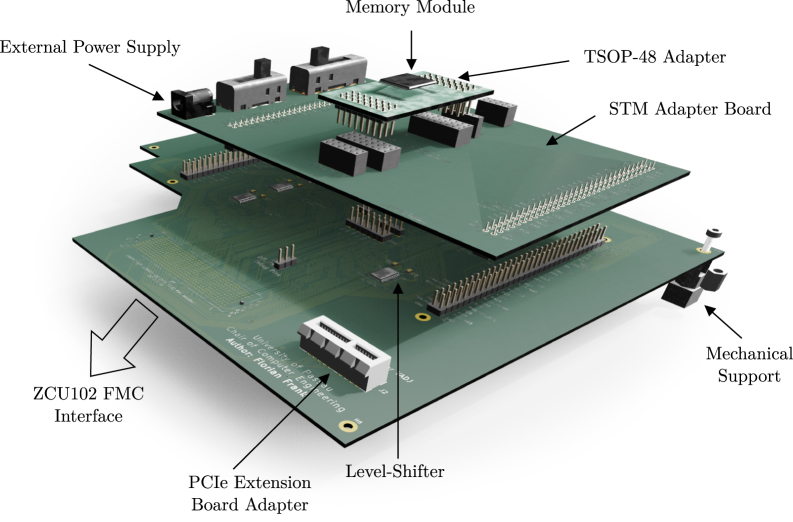
.

**(b):**
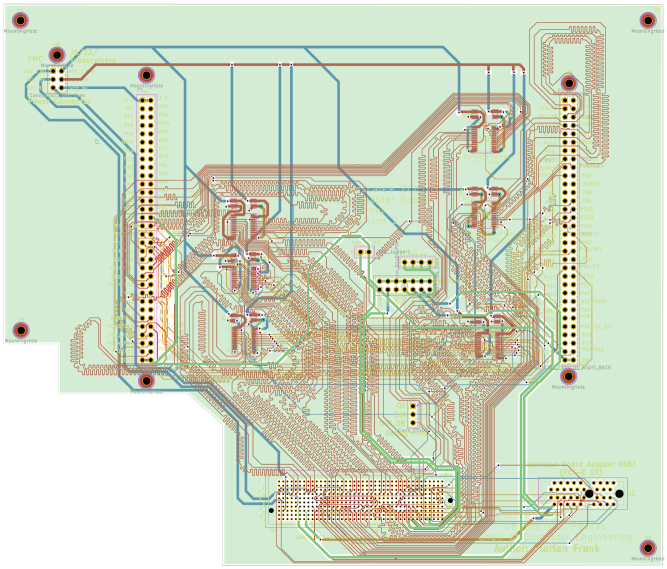
.

In addition to aligning the physical wiring between the evaluation platform and the memory modules, this board also matches the electrical characteristics of the connected memory module to those of the ZCU102. The pins on the FMC interface do not provide a fixed supply voltage; instead, the required voltage is negotiated with the connected FMC adapter board or configured manually via the System Controller User Interface of the board, as implemented in this work. However, the $V_{\text{adj}}$ power rail only supports voltages of 1.2 V, 1.5 V, and 1.8 V, and thus, the single-ended General Purpose Input/Output (GPIO) pins of the adapter are not compatible with the CMOS 3.3 V logic levels, which are required to interface with most of the memory modules used in this work.

To solve this issue, voltage level transition is implemented using TI SN74AVC8T245 [7] level shifters, soldered on the adapter PCB. These components enable the conversion between the 1.8 V and 3.3 V logic levels, with a constant delay of around 2.5 ns. Overall, this approach provides the flexibility to adjust the I/O voltages forwarded to the memory within a range of 1.2 V to 3.6 V by decoupling the power supply and integrating external measurement devices, such as the Siglent SPD1305X, which can be automatically controlled by our framework. Especially important for timing variation tests, the adapter PCB connected to the FMC connector ensures equal line lengths of the address and data lines, thereby minimizing timing distortions. Additional measures were implemented, such as separating the data lines with a dedicated ground layer to prevent crosstalk and routing all stable reference voltages on the backside of the PCB.

**Fig. 2:**
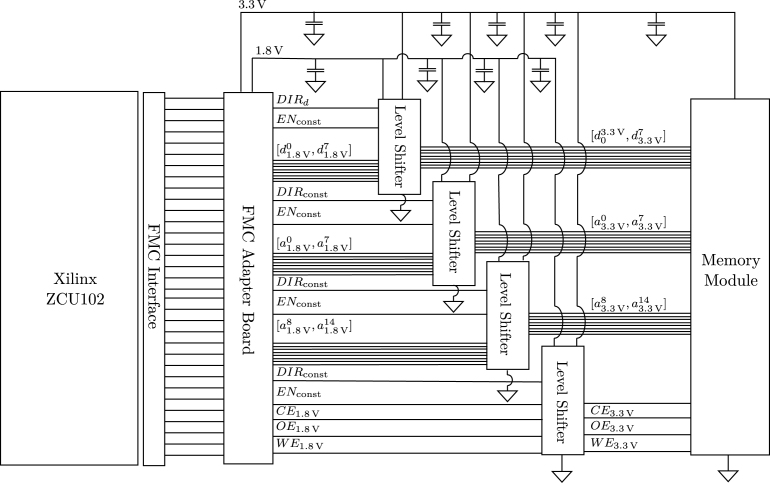
Physical wiring between the Zynq® UltraScale+^TM^ ZCU102 evaluation board and a memory module with a 15 bit address bus and 8 bit data bus. Four level shifters are used to convert logical signals between 1.8 V (supported by the board) and 3.3 V (supported by the memory module).

The schematic of this experimental setup with a memory module attached to a Zynq® UltraScale+^TM^ ZCU102 evaluation board is visualized in Fig. 2.

The level shifter requires additional control lines provided by the FMC adapter board to manage the transmission direction, either from the FPGA to the memory or vice versa. The $DIR_{d}$ wire specifies the direction of the data lines $\left[d^{0},d^{7}\right]$, while the address lines $\left[a^{0},a^{14}\right]$, as well as the Chip Enable ($\bar{CE}$), Output Enable ($\bar{OE}$), and Write Enable ($\bar{WE}$) signals, operate in one direction. For these signals, $DIR_{\text{const}}$ is assigned a constant value. Furthermore, a dedicated control wire is used to enable the level shifter during experiment execution. The design shown in Fig. 1(b) supports up to 16 data lines and 24 address lines, and provides additional control signals such as the Upper Byte Select ($\bar{UB}$), Lower Byte Select ($\bar{LB}$), and Sleep ($\bar{ZZ}$) pins, which are required by certain memory modules under test. Finally, to enable measurements with external power supplies, the power supply pins of the connected FPGA or microcontroller can be decoupled.

### 2.2 FPGA-based implementation

To enable a variety of PUF experiments on different memory modules, a platform is required that can precisely control the address and data buses in synchronization with the control signals ($\bar{CE}$, $\bar{OE}$, and $\bar{WE}$). Moreover, these I/O operations must be executed with fine timing granularity, requiring control at high clock frequencies, particularly for experiments such as row hammering or timing protocol violation. To implement an experimental setup that meets these requirements, we leverage the advantages of FPGAs, which provide precise timing control and prevent side effects such as caching through the implementation of a custom hardware design.

For our experiments, we use a Zynq® UltraScale+^TM^ ZCU102 evaluation board, with the memory modules connected via one of its FMC interfaces. The connection is established through the adapter board and circuitry as described in Section 2.1. The pins routed to the adapter board are specified as constraints, accessible through the custom FPGA-based PUF evaluation platform. All of these pins use the LVCMOS18 I/O standard. In order to support various memory modules, the communication protocol’s data bus width |d| and address bus width |a| are configurable. Additionally, any timing parameters of the SRAM-compatible memory controller are dynamically adjustable without requiring re-synthesis or reconfiguration of the FPGA.

**Fig. 3:**
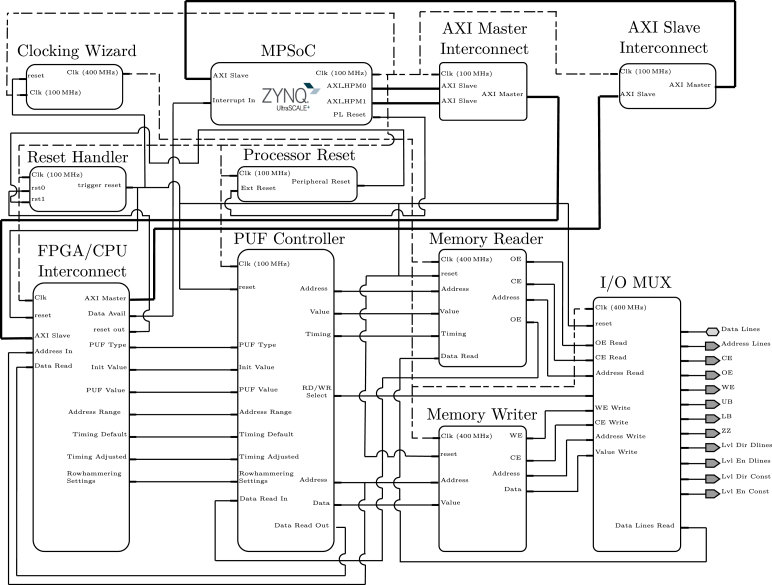
Simplified block design of the FPGA memory evaluator. The I/O pins forwarded to the FMC adapter board are shown on the right side.

This flexibility removes the need to recreate the bitstream for each experiment, enabling faster execution of multiple experiments across a broad parameter space. To allow quick architectural modifications, for example, replacing the parallel SRAM-compatible interface with a SPI interface, we designed the implementation in a modular manner, as depicted in the block design in Fig. 3.

The design consists of different components, implemented as dedicated Intellectual Property (IP) cores or Verilog modules. For example, the memory controller is divided into two modules: one to perform write operations (*Memory Writer*) and another one for read operations (*Memory Reader*). The *PUF Controller* is accepting a provided challenge and controlling the memory reader and writer modules to perform the PUF experiment on the connected memory module. In Fig. 3, the challenge parameters are shown on the left side of the *PUF Controller* module. These include the initialization value, the value to be written during PUF execution, the address range, and the memory’s default timing parameters applied during initialization. Furthermore, the challenge includes test-specific parameters such as adjusted timing settings for write and read latency variations or specific parameters to perform row hammering experiments.

As described above, the memory is interfaced through I/O ports, including the address and data lines, as well as the control signals routed to the FMC adapter. These ports are shown on the right side of Fig. 3. The remaining I/O pins are used to control the voltage level shifters, specific $\bar{UB}$, $\bar{LB}$, or $\bar{ZZ}$ pins.

An additional module, named *I/O MUX*, is required to select the memory address for either the *Memory Reader* or *Memory Writer*, depending on the mode of operation. It also sets the enable signal and controls the direction of the level shifters while placing the data lines in high impedance when reading from the memory.

To achieve a high timing resolution, the two modules comprising the memory controller are driven by higher clock frequencies than the rest of the hardware design. These clock frequencies are accomplished using the Xilinx Clocking Wizard IP-core [8], which generates higher clock frequencies from a slower base clock source of 100 MHz. For the experiments conducted in this work, the memory controller operates at a clock frequency of 400 MHz, allowing precise adjustments of every timing parameter in increments of 2.5 ns. An additional constant offset of approximately 2.5 ns is introduced by the level shifters attached to the FMC adapter, as explained in Section 2.1. This allows the timing parameters of the SRAM protocol to be configured in 2.5 ns steps. We do not provide a SPI interface for this test setup, as experiments using these interfaces can be performed on the previously described microcontroller-based setup, which provides sufficient accuracy at 120 MHz. Most memory modules supporting SPI interfaces do not exceed 50 MHz [9], [10], [11], while individual memories may reach up to 108 MHz. Higher frequencies typically result in no response from the SPI controller. Consequently, performing SPI experiments on the ZCU102 offers no practical advantage.

As explained below, we use the Advanced eXtensible Interface (AXI) bus [12] to interconnect the FPGA with one of the MPSoC’s CPUs, which handles the communication with the scheduler, explained in Section 2.2. However, this protocol supports only clock frequencies of up to 333 MHz. Consequently, the remaining design is driven by the default clock provided by the processing system with a frequency of 100 MHz, ensuring seamless synchronization between the two clock domains within the memory controller modules.

To interface the FPGA-based setup with the experiment scheduler described in Section 2.2, experiment configurations must be received, and measurement data must be transferred to the scheduler over the network. This connectivity is established through a standalone program, executed on the MPSoC’s Application Processing Unit (APU), specifically on the first core of the Arm® Cortex^TM^-A53. On the APU, parameter sets (including the PUF challenge C) are received as IP packets, transformed into a reduced custom format, and forwarded to the FPGA. To transfer data between the APU and the FPGA, we implemented a *FPGA/CPU Interconnect* module, which utilizes AXI-Lite to transfer the read data from the *PUF Controller* to the APU.

To run an experiment, a challenge C is transmitted from the APU to the FPGA, where it is received via the *FPGA/CPU Interconnect* module. This module contains a parser that processes the PUF type, address range, timing parameters, and other relevant data and forwards them to the *PUF Controller*, which controls the execution of the requested experiment. Subsequently, each experiment returns a tuple for every address in the address range $\left[a_{0},a_{n}\right]$ via the AXI interface to the CPU. Each tuple consists of the corresponding address and its associated read value. On the APU, the data is accessed from a shared memory segment within a callback function, which is invoked via a dedicated interrupt line triggered from the FPGA.

During the processing of the address–data tuples, each tuple is extended with a checksum, resulting in single data frames f≔{a,d,hash(a,d)}. These frames are then transmitted from the APU to the central experiment scheduler, where the integrity of the measurement data is verified using the hash value. The verified data is persistently stored and enriched with metadata to enable subsequent analysis.

### 2.3 Experiment scheduler and evaluation platform

**Fig. 4:**
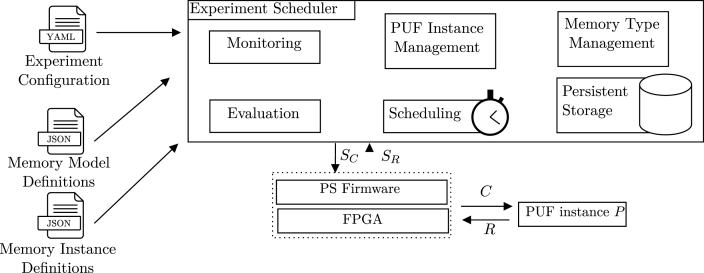
Overview of the structure of the PUF evaluation platform and scheduler for characterizing various types of memory-based PUFs.

Ensuring the reproducibility of measurement results, their persistent storage in a uniform format, and their enrichment with metadata is very important for the reliable evaluation and comparison of proposed PUF constructions. To achieve this, PUF experiments must be defined in a standardized format, enabling automated scheduling and monitoring of test executions on the ZCU102. Additionally, information about the PUF instance P under test, the applied challenge parameters $S_{C}$, and the corresponding measurement data must be stored consistently in a unified format. All this functionality is encapsulated in our experiment scheduler and evaluation platform. Its components and interconnection with the ZCU102 or microcontroller-based PUF execution platform are visualized in Fig. 4.

This Python program allows reading various configuration files that describe the properties of memory modules, such as their address and data bus widths or their timing parameters. Based on the description of specific memory models, individual memory instances can be defined to enable the assignment of measurements to a particular memory instance, while maintaining a record of all experiments executed on this module. All this information can be parsed during the scheduler’s startup and is persistently stored in a SQLite database. By simply adding additional configuration files, the database is automatically updated, allowing seamless integration of new memory models and instances.

An additional configuration file in YAML format specifies the test execution and enables the definition of experiment collections. A short example of such a test collection is depicted in Listing 1. In this example, a test collection is defined to be executed on a Rohm MR48V256C memory module, where the specific memory instance under test has the identifier FeRo1. The experiment collection consists of a reliability test, which first writes the value 0x55 to each address of the memory and then 0xaa, and subsequently verifies whether the values are read back correctly for each address. Next, read latency tests are performed in which the $t_{\text{oed}}$ timing parameter is modified, while all other timing values remain their default values. Here, an experiment for each value between 75 ns and 27.5 ns in 2.5 ns increments is generated and scheduled. The timing and reliability tests are executed ten times to assess the reproducibility of the measurements.

**Figure:**
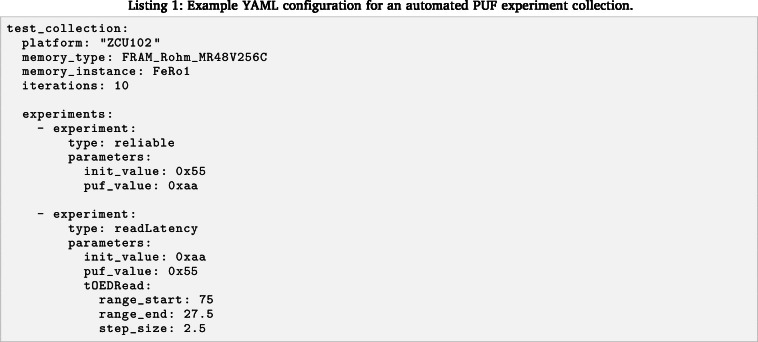

**Fig. 5:**
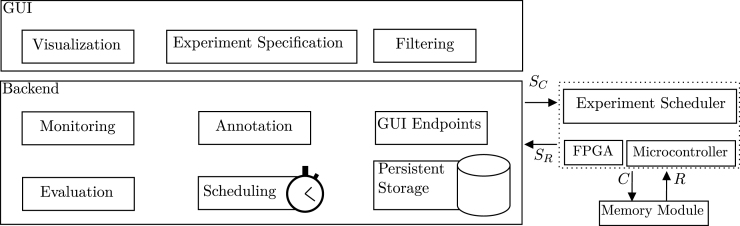
Second mode of operation of the scheduler, in which it runs as a microservice, connecting to backend and frontend components to enable experiment scheduling and monitoring.

To further lower the entry barrier, the experiment scheduler provides a second mode of operation, illustrated in Fig. 5. In this mode, the scheduler is deployed as a microservice that connects to a backend responsible for configuring, scheduling, and monitoring individual experiments. To this backend a graphical user interface can be attached, which allows users to submit an experiment configuration to the microservice scheduler and to monitor the experiment execution. The full evaluation is intended to be performed in the backend and subsequently visualized in the GUI; however, this functionality is currently under development. The GUI and backend implementation are available at.^1^ After parsing all configuration files or receiving a set of challenge parameters $S_{C}$ from the backend, the platform establishes a network connection either to the ZCU102 via Ethernet or to the STM32F429-DISC0 via a Universal Asynchronous Receiver Transmitter (UART) interface. The experiments are scheduled within a dedicated thread running a single-loop scheduler, while a second thread handles the reception of measurement data from either the ZCU102 or the STM32F429-DISC0. The scheduler transmits TCP packets to the firmware running on the Cortex^TM^-A53 of the ZCU102, whose payload, including the PUF challenge C, is encoded in JSON format. The firmware indicates when the experiment is started and responds with measurement data during experiment execution. The Python-based scheduler stores the received data in CSV files and adds corresponding entries to the SQLite database, with each record referring to the CSV file containing the associated measurement data. In case of the microservice-based operation mode, the retrieved responses $S_{R}$ are transmitted to the backend.

As shown in Fig. 5, the implementation is complemented by a GUI realized as a web application that communicates with the backend via REST endpoints and WebSockets. The current implementation supports the definition, scheduling, execution, and monitoring of experiments. However, as described above, functionality for visualizing and analyzing measurement results directly within the Graphical User Interface (GUI) is still under development.

### 2.4 Database scheme

**Fig. 6:**
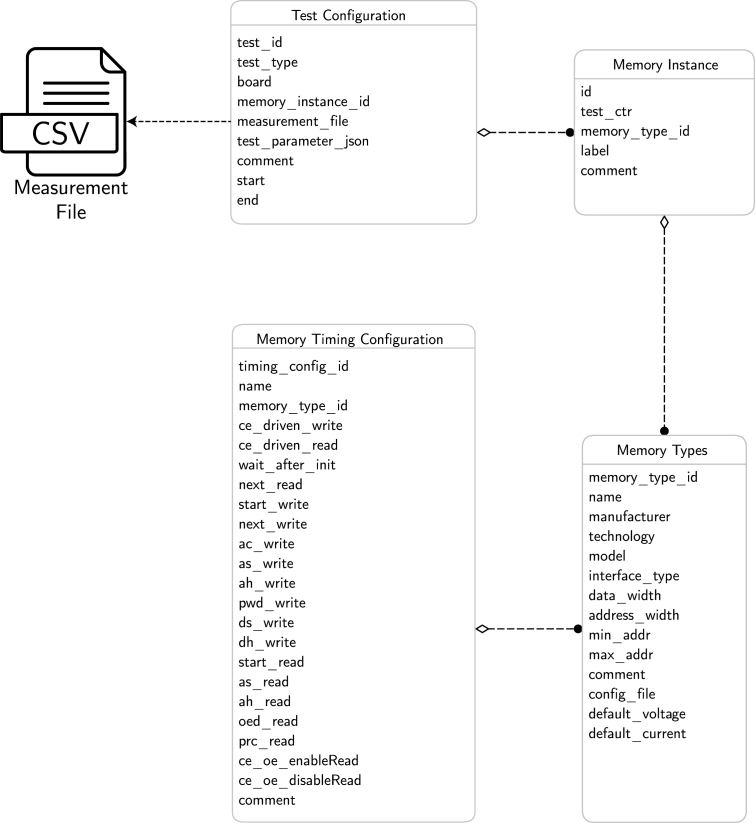
ER diagram showing the different tables of the developed database schema for storing memory models and instances, as well as metadata of the performed experiments. The resulting measurements are stored in dedicated CSV files.

As described earlier, our experiment scheduler stores metadata in an SQLite database, while the resulting measurement data is stored in easy-to-maintain CSV files. Each CSV file contains one entry per read address, including the corresponding read value and an additional checksum to detect potential data integrity issues. In Fig. 6, an Entity-Relationship (ER)-diagram of the database schema is visualized. In this schema specific memory types are stored in table *Memory Types*, for example a specific FRAM memory model. Each model is assigned a set of timing parameters (maintained in table *Memory Timing Configuration*), representing the default values extracted from the memory datasheets. The *Memory Types* table serves as template to generate multiple memory instances, which can then be used for experimental evaluation. The *Memory Instance* table also tracks the number of experiments performed on each module, enabling the detection of early signs of aging-related degradation. The *Test Configuration* table records each executed experiment, with each entry linked to a corresponding memory instance. It stores a reference to the measurement data, which is externally stored in a CSV file.

The parameters for experiment execution are defined within the test_parameter_json field in JSON format. This field contains the initialization and PUF values, as well as voltage and timing modifications relative to those stored for the memory instance in the *Memory Timing Configuration* and *Memory Types* tables. Further information, including sample data for each table, can be found in the scheduler’s documentation within the Zenodo repository, linked to this paper.

## 3 Design files summary

As described in detail in Section 2, our architecture consists of four components: the PCB adapter boards, the FPGA design implementing the PUF experiments, and the MPSoC firmware, serving as a bridge between the FPGA design and the last component, the experiment scheduler. Furthermore, these components can be categorized into hardware design files (PCB design and FPGA design) and software components (MPSoC firmware and experiment scheduler). The repository associated with this paper follows this distinction, as shown by the design files listed below:

• The **PCB** designs can be found in the hardware/pcbs directory. This folder contains the design files listed in Table 1, including the schematic and PCB layout files generated by KiCad for manufacturing, Gerber files for production and the mechanical support files to produce the FMC adapter board.
• In addition, **supporting software** is located in the software folder. Here, two additional subfolders ps_software and experiment_scheduler can be found. The first one implements the network functionality and the interaction with the FPGA executed on one of the MPSoC’s CPUs as described in Section 2.2. The design files are listed in Table 2.

• The **FPGA hardware design** is provided in the hardware/fpga_design directory. It includes all source files necessary to synthesize and implement the FPGA design, including the block logic that integrates all modules into a single design. The directory also includes scripts to automate the build process, as well as the constraints file for mapping logical signals to the pins of the FMC adapter board. Finally, simulation files are provided to test the hardware design in isolation. The design files corresponding to the FPGA implementation are listed in Table 3.
• The second folder, experiment_scheduler, contains all design files related to the experiment scheduler, which are listed in Table 4.

## 4 Bill of Materials summary

Focusing on the components placed on the PCB, this section presents the Bill of Materials (BOM) listed in Table 5. The prices shown are based on those offered by common electronic suppliers at the time of submitting this paper. The production of the adapter PCB itself, manufactured as a four-layer PCB with standard Hot Air Solder Leveling (HASL) surface finish was about 130€. This results in a total manufacturing cost of only about 179€ ($208). The ZCU102 evaluation board is priced at 2785€, leading to overall costs of 2964€. Our board is designed as a plug-in module, allowing the replacement of the ZCU102 with any other MPSoC that provides an FMC interface, potentially at the cost of limited timing accuracy. The ZCU102 is not strictly bound to this application and can be used independently of the measurement setup, offering an attractive cost-to-utility ratio. Additional details on each component in the BOM, including their reference on the PCB, and direct links to purchase the component, can be found in the repository corresponding to this paper.

**Table 1:** Design files corresponding to the PCB adapter board.

File	Description	Version	License	File location
fmc_memory_adapter.kicad _sch	KiCad schematic file for the FMC memory adapter.	v2.1	CERN OHL v2-P	fmc_memory_adapter/
fmc_memory_adapter.kicad _pcb	KiCad design file for the FMC memory adapter, implementing the physical interface between the FPGA and the memory module.	v2.1	CERN OHL v2-P	fmc_memory_adapter/
fmc_memory_adapter _gerber.zip	ZIP archive containing the Gerber files required for direct manufacturing.	v2.1	CERN OHL v2-P	fmc_memory_adapter /export
footprints	Collection of footprints and schematics for the PCB design; **some files require manual download as noted in the README files located within this folder.**	–	CERN OHL v2-P	fmc_memory_adapter/ footprints
pcb_mechanical_support .stl	3D-printed mount for the PCB, providing stable support and precise alignment.	v2.1	CERN OHL v2-P	fmc_memory_adapter/ pcb_mechanical_support
bom.csv	List of components used in this design, identical to Table 5.	v2.1	CERN OHL v2-P	fmc_memory_adapter/ bill_of_materials
doc/	Detailed documentation of the PCB.	v2.1	CERN OHL v2-P	fmc_memory_adapter/ doc

**Table 2:** Design files corresponding to the Cortex™-A53.

File	Description	Version	License	File location
build.sh	Script to automatically build the firmware. After execution, the firmware, exported as Executable and Linking Format (ELF)-file can be found next to the build script.	v2.1	Apache 2.0	scripts
flash.sh	Deploys the firmware (memory_evaluator.elf) to the Cortex^TM^-A53.	v2.1	Apache 2.0	scripts
CMakeLists.txt	To build the firmware, the original Vitis project was extracted and converted into a CMake-based build setup that is easy to compile and maintain. Further details are described in the project’s README.md and Section 5.	v2.1	Apache 2.0	.
xilinx_cortex_a53.cmake	Toolchain file loaded by CMake that defines the cross-compilation toolchain, including compiler and linker flags, for building the firmware targeting the Cortex^TM^-A53.	v2.1	Apache 2.0	toolchain
app_config.h/c	Configuration files that define IP addresses, ports, operation modes, and buffer sizes.	v2.1	Apache 2.0	src/config
board_support/	Basic platform functionality generated by Xilinx, containing platform-specific definitions and interfaces.	v2.1	Apache 2.0	src/config
src/	All source files of the firmware. Further descriptions of each file can be found in the project’s README.md and in the code documentation.	v2.1	Apache 2.0	src/config

**Table 3:** Design files required for the FPGA-based hardware design.

File	Description	Version	License	File location
main_block_design.tcl	The central block design is shown in Fig. 3. This design integrates all Verilog components into the final architecture and defines the connections to the constraints applied to the FMC adapter board.	v2.1	Apache 2.0	block_designs
axi_verification.tcl	A dedicated block design used to simulate the entire system, to receive data via AXI from the CPU, execute experiments and forward the measurement data to the CPU.	v2.1	Apache 2.0	block_designs
constraints.xdc	The constraints file defines the physical mapping of the FPGA’s ports to the predefined pins on the FMC interface accessible via the PCB adapter board.	v2.1	Apache 2.0	constraints
scripts/	This folder contains all scripts to create the project, generate the bitstream, export the hardware, and flash the programmable logic. It contains bash files that execute TCL scripts performing the requested actions. A detailed description of each script is available in the README.md file located in hardware/fpga_design.	v2.1	Apache 2.0	scripts
memory_read_top_module.v	Verilog top module implementing the SRAM-compatible read operation, allowing adjustable timing. This module is used for synchronization and control tasks.	v2.1	Apache 2.0	src
read_sram_protocol.v	Module implementing memory access by controlling the $\bar{CE}$ and $\bar{OE}$ signals and setting the address and data bus for read operations.	v2.1	Apache 2.0	src
memory_write_top_module.v	Verilog module implementing the SRAM-compatible write operation, allowing adjustable timing. Unlike the read module this module performs the entire write process.	v2.1	Apache 2.0	src
multiplexer.v	Verilog module implementing the *I/O MUX* as described in Section 2.2.	v2.1	Apache 2.0	src
puf_controller.v	Verilog module implementing the *PUF Controller* as described in Section 2.2.	v2.1	Apache 2.0	src
reset_handler.v	Verilog module implementing proper reset behavior for the two clock domains (100 MHz and 400 MHz).	v2.1	Apache 2.0	src
ps_pl_interface/	Folder containing all custom Verilog files and modules implementing the AXI-Lite-based data exchange between the CPU and the FPGA. Furthermore, it also includes the parser for the received experiment configurations.	v2.1	Apache 2.0	ips
sim/	Folder containing all test benches for verifying the design modules.	v2.1	Apache 2.0	sim

**Table 4:** Design files of the experiment scheduler.

File	Description	Version	License	File location
requirements.txt	List of all required Python packages, which can be installed using pip.	v2.1	Apache 2.0	.
setup_db.bash	Script to install all Python packages listed in requirements.txt.	v2.1	Apache 2.0	scripts
run.bash	Script to launch the application using the sample configuration located in the config_files folder.	v2.1	Apache 2.0	scripts
run_microservice.bash	Script to launch the application in microservice mode (required to start the GUI).	v2.1	Apache 2.0	scripts
endpoint_simulator.bash	Allows to simulate the MPSoC endpoint in order to test the scheduler in isolation.	v2.1	Apache 2.0	tests
sample_experiment_ write_latency_fram_lapis.yaml	Sample test configuration for performing write latency experiments on a FRAM memory module.	v2.1	Apache 2.0	samples
sample_experiment_ voltage_variations_mram.yaml	Sample test configuration for performing voltage variation experiments on an MRAM memory module.	v2.1	Apache 2.0	samples
src/	Experiment scheduler source files. More information can be found in the project’s README.md.	v2.1	Apache 2.0	src

**Table 5:** Bill of Materials.

Type	Manufacturer	Component number	Supplier	Unit price	Qty	Total price
FMC adapter	SAMTEC	ASP-134488	Mouser	32.11 €	1	32.11 €
Dual-supply bus transceiver	Texas Instruments	SN74AVC8T245PWG4	Mouser	1.32 €	6	7.92 €
Capacitors	KEMET	C0805C104K5RAC7411	Mouser	0.10 €	12	1.20 €
Pin headers 2 × 32	MPE	087-2-064-0-S-XS0-1260	Reichelt	1.10 €	2	2.20 €
Pin headers power supply	Würth Elektronik	613 006.1121	Mouser	0.32 €	1	0.32 €
Pin headers ground selectors	Würth Elektronik	610 314.1121	Mouser	0.81 €	2	1.62 €
Pin headers external ground	Amphenol Commercial Products	613 004.1121	Mouser	0.15 €	1	0.15 €
Pin headers ground selectors	Amphenol Commercial Products	G800NA306018EU	Mouser	0.09 €	1	0.09 €
Debug pin header	Würth Elektronik	613 003.1121	Mouser	0.10 €	1	0.10 €
Voltage/Ground selectors	MPE	149-1-002-F0-XS	Reichelt	0.10 €	8	0.80 €
Extension board socket	Amphenol	10018783-10010TLF	Mouser	0.70 €	1	0.70 €
Distance spacers M3	ECON	D3X08I5MT	Reichelt	0.11 €	2	0.22 €
Screws M3 thread	APM HEXSEAL	RM3X8MM-2701	Mouser	0.45 €	2	0.90 €

## 5 Build instructions

As described in Section 2, our proposed solution is built upon three components: first, a custom PCB design for physical interfacing; second, the implementation of hardware and software for the memory experiment platform on an MPSoC; and third, the software used to define, schedule, and evaluate experiments. The build instructions are organized according to this structure, starting with the PCB design.

### 5.1 Building the PCB adapter board

All files corresponding to the PCB layout, including the necessary schematics, the layout, footprints, and the BOM, can be found in the project repository within the folder hardware/pcbs/fmc_memory_adapter, as described in Table 1.

The adapter board PCB was designed using the open-source software KiCad.^2^ It is organized into two primary files: the schematic file fmc_memory_adapter.kicad_sch, which defines the connections between various pin headers, interfaces, level shifters, and capacitors; and the layout file fmc_memory_adapter.kicad_pcb, which is generated based on the schematic and contains the actual PCB layout. In case design changes are necessary on the PCB, these two files must be edited, and Gerber files for manufacturing must be manually exported. If the PCB is used directly, the corresponding Gerber files are available in the project’s repository, packaged in a ZIP archive named fmc_memory_adapter_gerber.zip. This format is accepted by most PCB manufacturers. The board should be fabricated as a standard four-layer PCB with a thickness of at least 1.6 mm. For our demo board, we selected a standard HASL surface finish. Due to the narrow wiring, a track width and spacing of 4 mils were selected. On the left side of the PCB, different voltage and ground connections must be established using 2.54 mm jumpers. By placing three jumpers horizontally on the voltage selector (J7), a voltage of 3.3 V is routed to the supply voltage connectors of the STM32 pin headers (P1 and P2). At the same time, the first reference voltage for the level shifters is set to 1.8 V, and the second to 3.3 V. For debugging purposes or when working with memory devices that use a different voltage standard, the second jumper can be removed. In this case, the $V_{CC}$ reference must be supplied by an external power source connected to one of the common ground pins (GND (J4)), found in the middle of the board. The ground connector (J1) must be bridged in order to establish a ground connection to the GND pins of the STM32 breakout pin headers.

Finally, for mechanical support, two stand-offs must be 3D-printed, provided within the file pcb_mechanical_support.stl. The support can be mounted to the PCB at mounting points MH0 and MH1 using M3 screws. A spacer with an internal thread can be inserted into each bracket to establish the mechanical connection, as shown in Fig. 1(a). Finally, this PCB can be connected to the FMC connector of the ZCU102, annotated as J8 on the adapter PCB and as J5 on the ZCU102. A more detailed documentation, guided by visualizations, can be found in the README.md located in hardware/pcbs/fmc_memory_adapter.

### 5.2 Building the MPSoC FPGA design and firmware

In this section, the preconditions and instructions to generate and flash the bitstream on the FPGA, as well as to compile the firmware for the Cortex^TM^-A53, are described.

#### Preconditions.

The hardware design included in the project repository targets Xilinx FPGAs and was therefore developed using the Xilinx Vivado 2022.2 IDE. All implemented modules are written in standard Verilog/SystemVerilog, making them portable to other FPGAs. However, some IP cores are specific to Xilinx devices. For instance, the Clocking Wizard LogiCORE IP^3^ is used to generate the required clock frequencies, or the IP cores to establish the AXI interconnects. Finally, the Zynq UltraScale+ MPSoC Processing System^4^ to configure the MPSoC’s Processing System is only available on Xilinx MPSoCs.

In order to generate the bitstream for the Zynq® UltraScale+^TM^ ZCU102 evaluation board, the *Vivado ML Enterprise Edition* is required. To compile the firmware for the Processing System (PS), the necessary toolchains must be installed, which come with Vitis^TM^ 2022.2. Furthermore, building the firmware requires Make and CMake to be installed.

#### Build instructions.

For easier use, we provide build scripts for each step of the build process, while the whole process of creating the project, generating the bitstream, exporting the hardware, compiling the firmware, and flashing the device can be executed by a single script setup_and_run_environment.sh, located in the repository’s root under scripts.

**Fig. 7:**
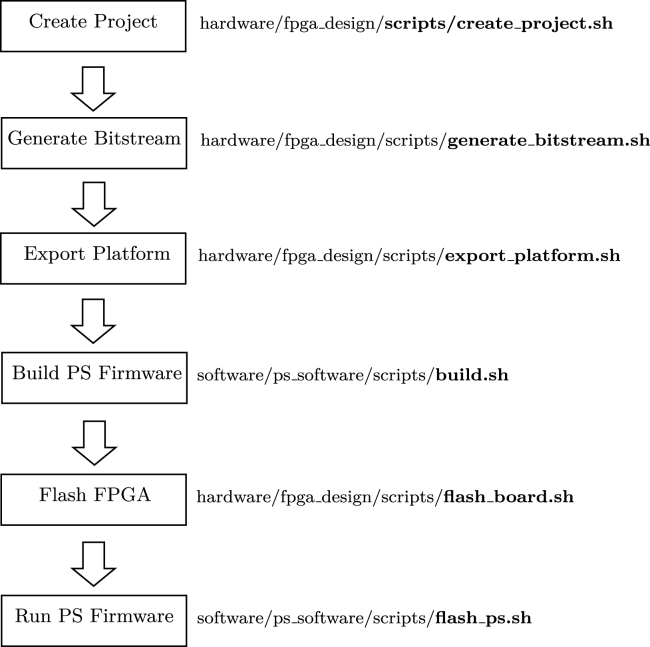
Build steps from creating the project to flashing the bitstream and firmware onto the ZCU102.

This script simply executes multiple bash scripts, which again run Tool Command Language (TCL) scripts using Vivado and Vitis in batch mode. The individual scripts can also be executed manually, as visualized in Fig. 7.

The individual scripts require adjustments if Vivado and Vitis are not installed on Linux in the default path under /opt/Xilinx. However, each of the scripts simply calls a TCL script, which can be executed manually. Beginning with the first script, create_project.sh, which creates a new Vivado project and imports all necessary design files and generates the block design and necessary Hardware Description Language (HDL) wrappers. The project is now located in the folder hardware/fpga_design/hardware/memory_evaluator folder and can be opened in Vivado, for example, to adjust the data and address bus widths. Alternatively, it can be directly built using the generate_bitstream.sh script, which runs all steps from synthesis to implementation and finally bitstream generation, and creates checkpoints between each step. After the bitstream is generated, the hardware can be exported using the export_hardware.sh script, which creates the required hardware description and include files for the Cortex^TM^-A53 firmware. After exporting the hardware, the Cortex^TM^-A53 firmware can be compiled using build_firmware.sh, which starts the CMake build process and generates the required ELF-file. Potentially, the path of the cross-compilation toolchain must be adjusted in the toolchain file, listed in Table 2.

### 5.3 Building the experiment scheduler

Since the scheduler is implemented in Python, no specific build steps are necessary. To run the scheduler, Python 3.10 or higher needs to be installed on the system. Furthermore, the required Python packages must be installed. This can be done automatically by running setup_db.bash located in the software/experiment_scheduler/scripts folder, which creates a virtual environment and installs all required packages listed in requirements.txt. Furthermore, it creates the initial SQLite database schema.

## 6 Operation instructions

First, the PCB adapter board must be connected to the ZCU102 board via the FMC (J5) connector, and a memory must be plugged into the PCB using the adapters as depicted in Fig. 1(a).

The generated bitstream and firmware can be flashed by first executing the flash_fpga.sh script to transfer the bitstream onto the FPGA and flash_ps.sh to program the Cortex^TM^-A53 with the generated firmware. To check the status of the firmware, a serial connection should be established to the PS before flashing using a serial terminal program, configured with a baud rate of 115 200, e.g., on /dev/ttyUSB0. The IP address to access the device is also shown here. This IP is distributed by Dynamic Host Configuration Protocol (DHCP) if a DHCP server is available; otherwise, it runs into a timeout and sets the default address to the IP stored in the variable SERVER_IP_ADDRESS within the app_config.h file, listed in Table 2.

Finally, the experiment scheduler can be started by executing run.sh located in software/experiment_scheduler/scripts. Before running the application, parameters such as IP addresses, ports, timeouts, and database names can be defined in src/utils/definitions.py. The run script launches the scheduler and loads a configuration file, which must be provided as a command-line argument, e.g. by running run.sh sample_experiment_write_latency_fram_lapis. yaml (when starting as a standalone application). The program will automatically establish a connection to the ZCU102 board and start executing the defined experiments, track the experiments in the SQLite database, and save the measurement results within the output_results folder. Finally, the measurement data can be queried from the database, and the corresponding measurement files can be parsed and evaluated. To run the program as a microservice that connects the GUI and the backend, the script run_microservice.bash must be executed. The backend and GUI can be started using Docker by running docker compose up. However, further information can be found in the corresponding repository.

## 7 Validation and characterization

In this section, we evaluate the timing guarantees of our setup and compare it to our previous implementation based on an STM32F429I-DISCO development board. This work focuses exclusively on a measurement setup that enables a systematic search for PUFs in emerging non-volatile memory technologies, which is still an open research topic. Therefore, we focus primarily on evaluating the experiment setup itself, rather than introducing a new type of PUF. For this reason, the experiments are not intended as full validation of the memory modules as PUFs. They rather demonstrate that our setup can generate behaviors leading to bit-flips on real non-volatile memory modules. A full validation of these memory modules according to PUF characteristics is still ongoing. Nevertheless, we validate correct operation by conducting read and write violation experiments on eleven LAPIS Semiconductor® MR48V256C FRAM memory modules.

### 7.1 Timing analysis

Before executing any experiment, our FPGA design was fully tested using simulations, allowing us to test the behavior from sending AXI-Lite commands from the PS to performing memory operations and sending back the measurement results. However, to ensure precise and reproducible measurement results, we analyzed the timing accuracy of our setup using a Keysight MSOX3104T oscilloscope, focusing in particular on the timing of the implemented memory controller. Specifically, the timing resolution of the read and write pulses on the ZCU102 with the attached FMC adapter was analyzed and compared with our initial evaluation platform based on an STM32F429-DISCO. The results are shown in Fig. 8.

These figures show the average pulse widths and the corresponding standard deviations of the three SRAM control signals $\bar{CE}$, $\bar{WE}$, and $\bar{OE}$, when cycling through different $t_{\text{prc}}$ and $t_{\text{pwc}}$ values configured in clock cycles, based on the frequency of the board’s memory controller. We analyzed the timing guarantees through repeated measurements of the electrical signals, based on the SRAM protocol with the three probes directly connected to the output pins on the FMC adapter board or the STM32F429I-DISC0. The frequencies applied to the memory controller of the STM32F429I-DISC0, allow timing adjustments in increments of slightly more than 8.33 ns, reflecting the 120 MHz frequency configured via its Phase Locked Loop (PLL). The supported pulse widths are shown in Fig. 8(a). Additionally, we analyzed the timing behavior of the data and address lines and observed an average standard deviation of 0.47 ns from the expected values. We could also confirm that the pinouts for the data and address buses returned results in the same range.Fig. 8Timing characteristics of the SRAM protocol pulse widths and standard deviations of the two evaluation platforms. (a) illustrates the available timing steps and corresponding standard deviations for the STM32F429I-DISC0. (b) shows the timing resolution and deviations measured on the Xilinx Zynq® UltraScale+^TM^ ZCU102 evaluation board.Fig. 8

**(a):**
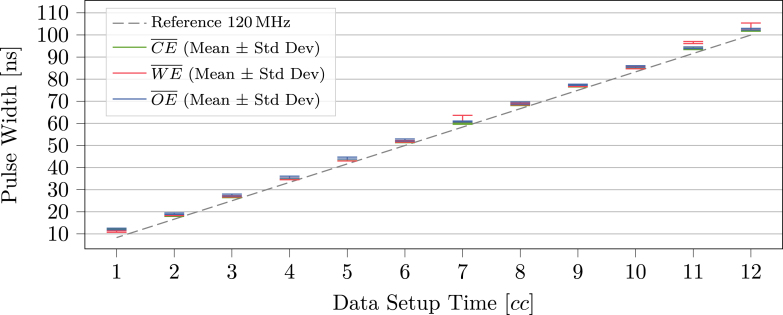
.

**(b):**
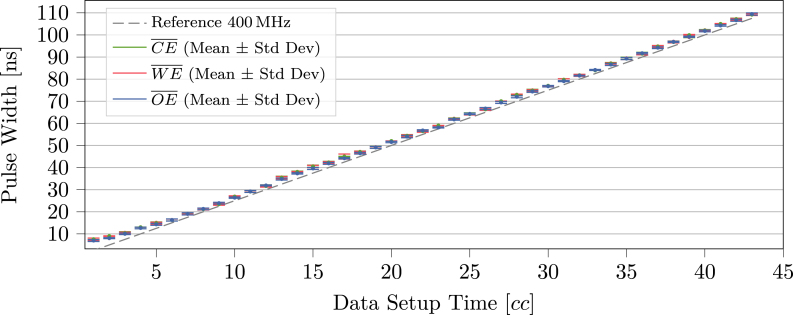
.

In Fig. 8(b), the possible pulse widths of the Xilinx Zynq® UltraScale+^TM^ ZCU102 evaluation board are shown, analyzed along the same range from 0 ns to 110 ns. On this platform, timing parameters can be adjusted in increments slightly above 2.5 ns, with a lower boundary of approximately 7.4 ns. This elevated boundary is due to the use of level shifters for adapting the logic levels of 3.3 V. Therefore, we have experimentally validated the delay of these devices by repeated measurements on the control signal pins with and without the level shifters and determined an average offset of 2.39 ns. These measurements were performed using a previous version of the FMC adapter board, where the level shifters could be measured in isolation. Additional information on this board is given in the repository in hardware/pcbs/fmc_memory_adapter. The remaining 5 ns are caused by one clock cycle for activating a control signal and one extra cycle for turning it off in a dedicated state of the state machine, controlling the memory access. This offset is constant, and afterward, the timing can be configured in steps of 2.5 ns. However, as demonstrated later in this section, timing values at the minimum boundaries of the evaluation platforms do not produce usable results on any of the tested memory devices. The control signals exhibited an average jitter with a standard deviation of 0.35 ns, slightly lower than the jitter observed from the STM32F429I-DISC0 board. However, it is expected that the newer PCB adapter board with improved signal integrity will further reduce this jitter. In order to achieve statistical significance, these values are evaluated on 5000 individual timing measurements for each experiment setup.

### 7.2 Validation of the functionality on LAPIS Semiconductor® MR48V256C memories

After simulating the behavior of our setup and conducting the timing and jitter analysis on the logical pins of the FMC adapter board, we validated the correct operation of our architecture by performing reliability tests, read and write latency violation experiments, as well as row hammering tests on eleven LAPIS Semiconductor® MR48V256C FRAM memory modules.

The memory module under test offers a memory capacity of 32 kB, accessible via an 8-bit data bus and a 15-bit address bus, utilizing an SRAM-compatible parallel interface supported by our setup. The recommended supply voltage range is 2.7 V to 3.6 V, and the $\bar{CE}$ to data access time is 70 ns.

After validating the correct operation by writing various initialization values to all addresses and subsequently reading back the expected values, we conducted write-latency violation experiments by first using our initial STM32F429I-DISC0-based setup to evaluate the characteristic behavior of the memory modules when applying write pulses of a different length.

**Fig. 9:**
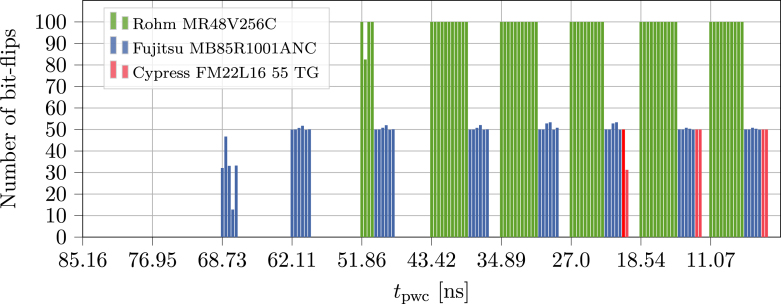
Number of bit-flips on FRAM memories, collected using an STM32F429I-DISC0 board. The labels of the x-axis follow the previously evaluated average pulse width durations.

**Fig. 10:**
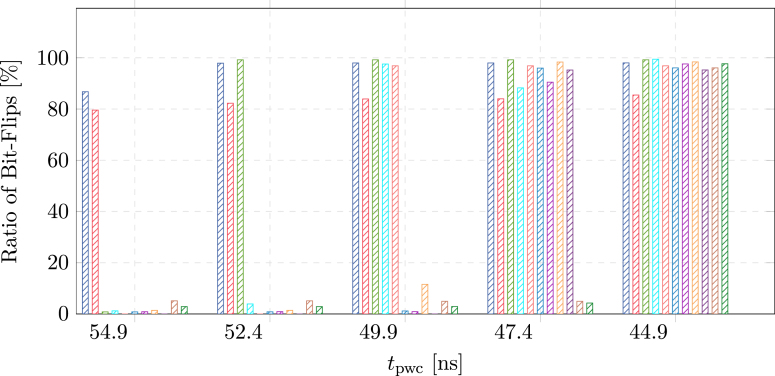
Visualization of the bitflip ratio across eleven LAPIS Semiconductor® MR48V256C modules evaluated along the range $t_{\text{pwc}}\in \left[43.42,62.11\right]\;ns$ using the Xilinx® Zynq UltraScale+^TM^ ZCU102 board.

When performing read or write latency experiments, the entire address space is initialized with an initialization value $d_{I}$, under timing aligned with the memory’s timing specification. Subsequently, the actual PUF experiment is performed. For write latency violations, we write $d_{\text{puf}}$, which is typically the binary inverse of $d_{I}$ under reduced timing conditions, where one timing parameter is modified. In the end, the entire memory content is read back under nominal timing. For read timing violation experiments, no second write operation is performed; instead, the read operation itself is executed with a single altered timing parameter.

Using the STM32F429I-DISC0-based setup, we first performed write-latency violation experiments by gradually reducing the $\bar{WE}$ pulse width, denoted as $t_{\text{pwc}}$. For all experiments, we use a checkerboard pattern with $d_{I}=0x55$ and $d_{\text{puf}}=0xaa$ to analyze the directionality of bit-flips. Initially, $d_{\text{puf}}$ could be written successfully; however, once a certain timing threshold is exceeded, no write operation with $d_{\text{puf}}$ succeeded, and the memory consistently returned the previous initialization value $d_{I}$. Since $d_{I}$ is the bitwise inverse of $d_{\text{puf}}$, this behavior corresponds to a 100% bit-flip rate. The results of these experiments are shown in Fig. 9.

The results show that when setting $t_{\text{pwc}}$ to 62.11 ns $d_{\text{puf}}$ can be read reliably across the entire memory space. Reducing $t_{\text{pwc}}$ by just one clock cycle on the STM32F429I-DISC0 (resulting in a $\bar{WE}$ pulse of 51.85 ns) caused four out of the eleven memory modules to produce a uniform response of 0x55 (with $d_{I}=0x55$ and $d_{\text{puf}}=0xaa$), indicating that no write operation occurred. One module exhibited a bit-flip rate of 82.52%, while two modules showed less than 1% of flipped cells. The remaining five modules continued their operation reliably. Further reducing $t_{\text{pwc}}$ to produce a $\bar{WE}$ pulse of 43.42 ns prevented all modules from returning the expected $d_{\text{puf}}$, and all cells consistently returned the initialization value.

These experiments demonstrate that the LAPIS Semiconductor® MR48V256C modules either operate reliably or fail to perform any write operation when the timing exceeds a certain threshold. However, the observation of bit-flips in a single memory module at a $t_{\text{pwc}}$ of 51.86 ns suggests that further bit-flips may occur when adjusting $t_{\text{pwc}}$ within the range of [43.42,62.11]ns with finer granularity. At this stage, a setup enabling higher timing resolution and flexibility was necessary, which the FPGA-based architecture presented in this work provides.

Fig. 10 shows the results of write-timing violation experiments, obtained using the Xilinx Zynq® UltraScale+^TM^ ZCU102 evaluation setup. In this plot, we clearly see the effects of reducing $t_{\text{pwc}}$ in smaller steps of 2.5 ns, leading to more and more memories to flip their bits. However, this plot also shows that even with the significantly enhanced timing resolution, no usable PUF behavior could be identified on the tested LAPIS Semiconductor® MR48V256C modules. While a few memory modules exhibit minor variability of up to 10%, the resulting outputs are random and not reproducible on the same device. Only two memory modules produce bit-flip rates in the range of 20%; however, these bit-flips also lack stability, preventing the extraction of hardware fingerprints. Row hammering experiments were also conducted on these memory modules, but no bit-flips were observed. This result is consistent with the expected behavior of FRAM technology, as no leakage effects are present, characteristic of DRAM memories.

However, these results primarily reflect the characteristics of the memory modules under test and do not present the derivation of PUF properties from individual modules. Nevertheless, by comparing the characteristics measured with the STM32 board with integrated memory controller to the higher-resolution results obtained from our MPSoC setup, we can confirm the correct functionality of our implementation. Our architecture further provides a foundation for systematically testing different memory modules, enabling the potential observation of PUF behavior in novel FRAM, MRAM, or ReRAM memory devices.

## 8 Conclusion

In this work, we demonstrated a fully automated evaluation platform for memory-based PUFs using a custom setup consisting of a PCB adapter board and an FPGA-based memory controller that allows flexible adjustment of all timing parameters. This setup enables researchers to automatically test different non-volatile memory modules for PUF behavior, a process that is very time-consuming and error-prone when performed manually. Finally, we presented an experiment scheduler for defining experiments, their automated execution, and the persistent storage of the corresponding measurement results. We validated the feasibility of performing experiments with a 400 MHz FPGA clock, enabling pulse widths with a granularity of 2.5 ns, and demonstrated the functionality of our platform by performing experiments on eleven LAPIS Semiconductor® MR48V256C FRAM memory modules. The goal was not to introduce a new type of PUF; rather, these experiments confirmed the functionality of our platform when compared to a memory controller-based setup.

## 9 Future work

A significant portion of the work is left for future research. Specifically, an in-depth verification of NVMs using our setup needs to be conducted. This includes experiments, for example, under varying supply voltages and access times. Furthermore, the results obtained from the NVM modules must be analyzed, especially with regard to their robustness under a range of environmental stress conditions, such as different operating temperatures.

Apart from the additional work required for the collection and evaluation of measurement data, the platform itself requires further development and additional features. One component which requires significant ongoing work is the graphical user interface. It currently supports the definition, scheduling, and monitoring of experiments. Functionality for analyzing measurement data directly within the GUI has not yet been implemented. Other areas for future work include conducting experiments with a different FPGA that can directly provide sufficient voltage levels to interface with the memory modules, thereby eliminating the delays and potential jitter caused by voltage level conversion. A third revision of the PCB adapter board should also be considered, using more layers in order to reduce trace lengths and cross-talk, further improving signal integrity.

## Glossary

$\bar{CE}$ Chip Enable.
$\bar{LB}$ Lower Byte Select.
$\bar{OE}$ Output Enable.
$\bar{UB}$ Upper Byte Select.
$\bar{WE}$ Write Enable.
$\bar{ZZ}$ Sleep.
**APU** Application Processing Unit.
**AXI** Advanced eXtensible Interface.
**BOM** Bill of Materials.
**DHCP** Dynamic Host Configuration Protocol.
**ELF** Executable and Linking Format.
**ER** Entity-Relationship.
**FMC** FPGA Mezzanine Card.
**FPGA** Field Programmable Gate Array.
**FRAM** Ferroelectric Random Access Memory.
**GPIO** General Purpose Input/Output.
**GUI** Graphical User Interface.
**HASL** Hot Air Solder Leveling.
**HDL** Hardware Description Language.
**IP** Intellectual Property.
**MPSoC** Multi-Processor System-on-Chip.
**MRAM** Magnetoresistive Random Access Memory.
**NVM** Non-Volatile Memory.
**PCB** Printed Circuit Board.
**PLL** Phase Locked Loop.
**PS** Processing System.
**PUF** Physical Unclonable Function.
**ReRAM** Resistive Random Access Memory.
**SPI** Serial Peripheral Interface.
**SRAM** Static Random-Access Memory.
**TCL** Tool Command Language.
**UART** Universal Asynchronous Receiver Transmitter.

## CRediT authorship contribution statement

**Florian Frank:** Writing – review & editing, Writing – original draft, Visualization, Validation, Software, Resources, Methodology, Investigation, Conceptualization. **Stefan Katzenbeisser:** Writing – review & editing, Writing – original draft, Supervision, Conceptualization.

## Ethics statements

This work did not involve human subjects or animals in its research.

## Declaration of competing interest

The authors declare that they have no known competing financial interests or personal relationships that could have appeared to influence the work reported in this paper.

## Footnotes

1 https://github.com/FlorianFrank/experiment_execution_hub.

2 https://www.kicad.org.

3 https://docs.amd.com/r/en-US/pg065-clk-wiz.

4 https://docs.amd.com/r/3.4-English/pg201-zynq-ultrascale-plus-processing-system/Zynq-UltraScale-MPSoC-Processing-System-v3.4-LogiCORE-IP-Product-Guide-PG201.
